# Preparation of the Tetrameric Poly(VS-St-BMA-BA) Nano-Plugging Agent and Its Plugging Mechanism in Water-Based Drilling Fluids

**DOI:** 10.1021/acsomega.2c02784

**Published:** 2022-08-03

**Authors:** Rongchao Cheng, Zhen Lei, Yang Bai, Jie Zhang, Huijun Hao, Gang Xie

**Affiliations:** † CNPC Engineering Technology R&D Company Limited, Beijing 102200, China; ‡ Southwest Petroleum University, Chengdu 610500, China

## Abstract

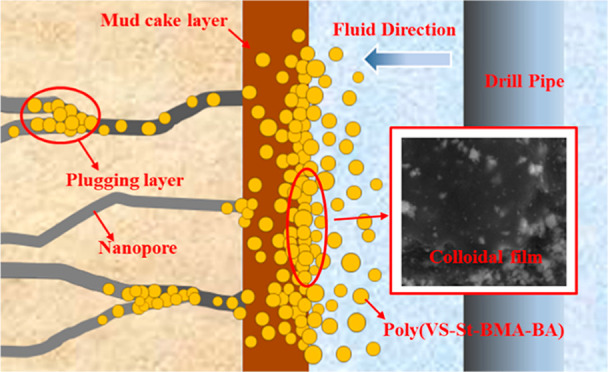

In response to the current problem that micron-scale plugging agents cannot effectively plug shale nanopores and fractures, tetrameric poly(VS-St-BMA-BA) nanoparticles were synthesized by the Michael addition reaction using sodium vinyl sulfonate, styrene, butyl methacrylate, and butyl acrylate as raw materials. The nanoparticles poly(VS-St-BMA-BA) were characterized by infrared spectroscopy, particle size analysis, and thermogravimetric analysis. The particle size distribution of poly(VS-St-BMA-BA) at room temperature ranged from 62.17 to 96.44 nm, with a median particle size of 75.8 nm, and could withstand high temperature of 359.5 °C. The effects of poly(VS-St-BMA-BA) on the rheological parameters of drilling fluid and the effects of different temperatures on the median particle size were investigated by the drilling fluid performance testing methods and high-temperature stability testing methods. The results showed that the apparent viscosity, plastic viscosity, yield point, and high temperature and high pressure water loss of drilling fluid gradually decreased with the increase in poly(VS-St-BMA-BA) dosage; when the addition of poly(VS-St-BMA-BA) was 2.0%, the overall performance of drilling fluid was better, the filtration loss was 4.4 mL, and the drilling fluid had good water loss wall building performance. The median particle size of poly(VS-St-BMA-BA) was 132.60 nm (the particle size at room temperature was 75.8 nm) after standing for 16 h at 180 °C, indicating that poly(VS-St-BMA-BA) has good high-temperature stability and dispersion stability. The plugging performance and plugging mechanism of poly(VS-St-BMA-BA) under extreme conditions (high temperature) were investigated by the plugging performance test method and pressure transfer method. The results showed that the plugging rate of artificial mud cake and artificial core reached 48.18 and 88.75%, respectively, when the amount of poly(VS-St-BMA-BA) was added at 2.0%. In the pressure-transfer experiments, poly(VS-St-BMA)-BA) could invade the 2 mm position of the nanopore fracture on the core surface and form a sealing barrier layer to prevent the further invasion of liquid. Combined with the pressure-transfer experiment, it shows that poly(VS-St-BMA-BA) can enter the nanopore and fracture at a certain distance under the action of formation pressure and keep accumulating to form a tight blockage, which can effectively prevent the filtrate from entering the nanopore fracture of the shale formation. Poly(VS-St-BMA-BA) is expected to be used as a promising nano-plugging agent in water-based drilling fluids.

## 1 Introduction

As a clean and efficient energy, shale gas is an effective way to solve the current energy shortage problem in my country. My country is rich in shale gas resources, with resource reserves as high as 134 trillion cubic meters, Sichuan is also a major province of shale gas, with resources accounting for 20.46% of the national total, and the shale gas resources in the Sichuan Basin have become the main development area of national energy development.^1−3^ With the increasing demand for shale oil and gas resources, the rapid development of the deep drilling fluid technology has provided strong support for the exploration and development of deep shale oil and gas. However, shale formations are mostly developed with natural pores and fractures,^4^ during the development of shale gas, there is still drilling fluid filtrate infiltrating into shale pores and fractures,^5,6^ which weakens the structural force of the wellbore and causes the instability problem;^7−9^ therefore, in order to improve the wellbore stability, it is necessary to strengthen the ability of water-based drilling fluid to plug microfractures.^10−12^ Adding effective nano-plugging materials to drilling fluids is the key to solving the problem of shale wellbore instability.^1,13^ There is a wide range of existing plugging agents, including ultra-fine calcium carbonate, fibers, asphalts, polymers, gel particles, and so forth. However, these plugging agents are too large to effectively plug nanoscale pores.^18^ Considering the size match, according to many studies, nanoscale plugging agents are critical for wellbore stability in troubled formations.^25^

In the deep development of shale gas formations, water-based drilling fluids are required to have excellent plugging performance for frequent wellbore instability problems.^14^ Huang et al. (2022) synthesized functionalized polystyrene latex (FPL) by micellar polymerization in order to solve the problem of borehole instability. In artificial mud cake with low permeability, FPL water suspension has good sealing performance. This study provides a good reference for the development of high-temperature NPAs (nano-plugging agents) and the establishment of NPAs evaluation methods. However, it is difficult to block filter paper and ceramic filter discs by themselves.^15^ Ma et al. (2020) pointed out that the poor dispersibility of multi-walled carbon nanotubes at high salinity and high temperature severely limits their application in the oil and gas extraction industry, by modifying multi-wall carbon nanotubes, two kinds of modified multi-wall carbon nanotube plugging agents, MWCNTs-g-SPMA-1 and MWCNTs-g-SPMA-2 were obtained, and the plugging agent can show good dispersibility in saturated brine and high temperature (170 °C) and excellent plugging performance for low-permeability reservoirs.^16^ Yu et al. (2020) synthesized NaSS-MMA copolymers by emulsion polymerization; the particle size of nanoparticles is mainly concentrated in 35–191 nm, which can resist high temperature of 347.6 °C and hardly change the rheological properties of drilling fluid. When the addition amount of NaSS-MMA nanoparticles in the solution is 0.5 wt %, the plugging rate can reach 86.1%, showing excellent plugging performance.^17^ Li et al. (2020) showed synthesis of polymer nanospheres with double cross-linked structure; the average particle size is 133 nm, and after high-temperature treatment at 150–200 °C, it can retain about half of the original particle size, and the initial decomposition temperature is about 315 °C, which can effectively seal the pores of shale. However, the plugging agent has a great influence on the rheological properties of the drilling fluid, and it is difficult to meet the technical requirements of the drilling fluid for the performance.^18^ Ma et al. (2019) used acrylamide, 2-acrylamido-2-methyl-1-propanesulfonic acid, *N*-vinylpyrrolidone, and modified nanosilica as raw material, the copolymer (PAAN-SiO_2_) was synthesized, and it can resist high temperature of 180 °C, which can significantly reduce shale permeability, prevent fluid intrusion, and improve wellbore stability. However, the size of the plugging agent is large and cannot effectively plug the nanopores and seams.^19^ Ma et al. (2019) described the poor dispersion of silica at high salinity and high temperature, and a modified silica (SiO_2_-*g*-SPMA) nano-plugging agent was synthesized by grafting anionic polymers on silica. SiO_2_-*g*-SPMA can be stable for 24 h at 170 °C and also stable in the weak alkaline environment. The plugging performance in water-based drilling fluid in low-permeability reservoirs reaches 78.25%, which can effectively prevent drilling fluid filtrate from invading the formation. However, the modified nano-plugging agent has insufficient temperature resistance and cannot be used in deep and ultra-deep wells with higher temperature.^20^ Liu et al. (2019) described shale gas wellbore instability and leakage, and a micro–nano polymer microsphere plugging agent was developed by emulsion polymerization, the plugging agent can form a continuous and dense plugging layer on the well wall through the mechanism of “adsorption-bridging-deformation filling” and “chemical plugging agent,” preventing pressure transmission and filtrate intrusion. Moreover, it has good rheological, filtering, and lubricating properties, which can effectively block shale micro- and nanoscale fractures, inhibit shale hydration, and provide drilling fluid technical support for shale gas drilling and development.^21^ According to Jia et al. (2016) in order to solve the problem of wellbore instability, achieving efficient development of shale gas, a nano-plugging agent NFD-1 was synthesized, NFD-1 can be dispersed in water and has a particle size of about 130 nm, which has little effect on the rheology of drilling fluid, and when the concentration is 5%, the plugging rate can reach 80%, it has a strong sealing performance.^22^

It was found that most of the plugging agents need to be improved in terms of particle size, strength, temperature resistance, and plugging effect. In this paper, poly(VS-St-BMA-BA), a water-based nano-plugging material, was synthesized by the Michael addition reaction under styrene, butyl methacrylate (BMA), and butyl acrylate (BA) as the main raw materials for sealing nanopore joints in shale formations. Poly(VS-St-BMA-BA), as a nanomaterial, can both enter the nanopore joints to form an effective seal and can be used in combination with large particles of rigid materials. The benzene ring in the structure of poly(VS-St-BMA-BA) gives it good temperature resistance. Poly(VS-St-BMA-BA) is expected to be an excellent nano-plugging agent for water-based drilling fluids to maintain well wall stability in shale formations and speed up the drilling cycle.

## 2 Results and Discussion

### 2.1 Infrared Spectrum

**Figure 1:**
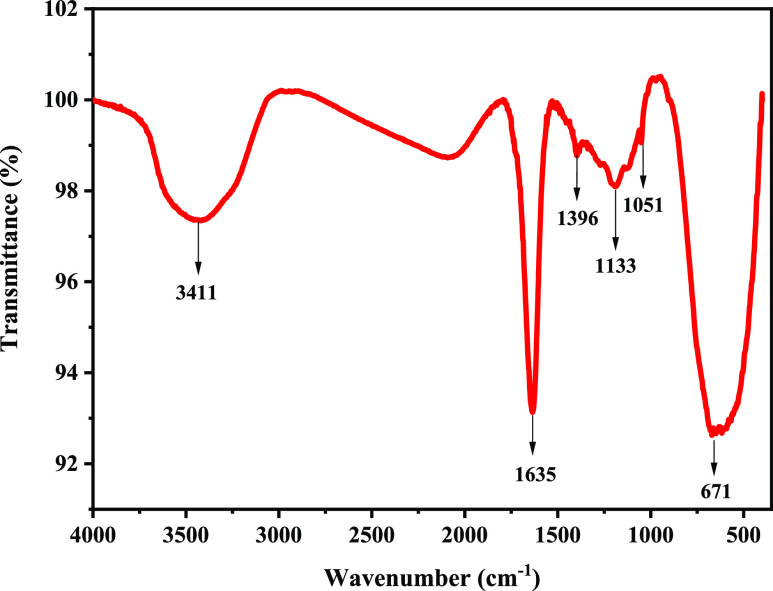
Infrared spectra of poly(VS-St-BMA-BA).

Figure 1 shows the infrared spectrum of the water-based nano-plugging agent poly(VS-St-BMA-BA). As can be seen in Figure 1, 3411 cm^–1^ indicates the stretching vibration peak of −OH of liquid water, and 1635 cm^–1^ indicates the stretching vibration peak of the benzene ring skeleton and the bending vibration peak of −OH of liquid water; the symmetric variable angle vibration peak of −CH_3_ is shown at 1396 cm^–1^, the antisymmetric stretching vibration peak of C–O–C and the asymmetric stretching vibration peak of S=O of sulfonate are shown at 1133 cm^–-1^, 1051 cm^–1^ is the symmetric stretching vibration peak of S=O of sulfonate, and 671 cm^–1^ is the external deformation vibration peak of C–H on the benzene ring.^23,24^ From the results of infrared spectra, poly(VS-St-BMA-BA) was synthesized successfully. Among them, styrene (St) is the polymerization monomer, and divinylbenzene (DVB) is the cross-linking agent, and they contain high-temperature-resistant benzene ring, which can enhance the temperature resistance of poly(VS-St-BMA-BA). Sodium vinyl sulfate (VS) as the polymerization monomer contains the sulfonic acid group, which can improve the salt resistance and hydrophilic performance of poly(VS-St-BMA-BA).

### 2.2 Particle Size Distribution of Poly(VS-St-BMA-BA) at Room Temperature

**Figure 2:**
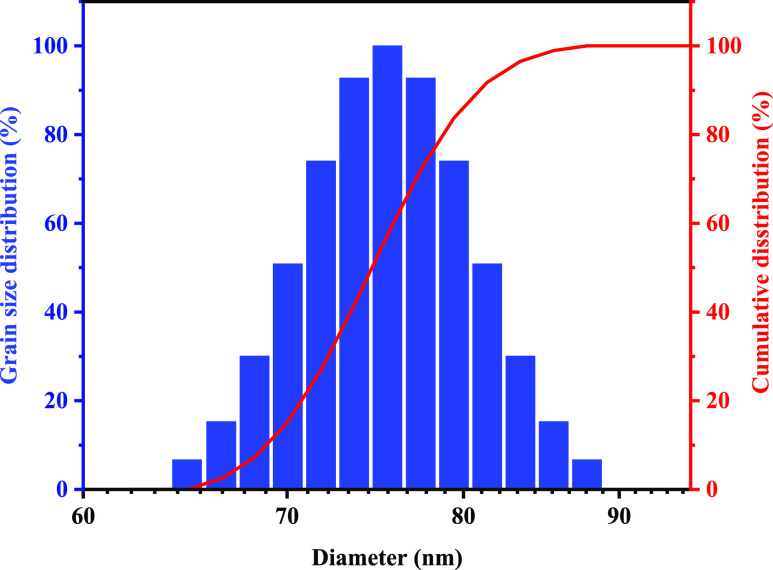
Poly(VS-St-BMA-BA) particle size distribution.

Figure 2 shows the particle size distribution of the water-based nano-sealant poly(VS-St-BMA-BA). As can be seen from Figure 2, poly(VS-St-BMA-BA) had a more concentrated particle size distribution with a spiky parabola, the particle size distribution is between 62.17 and 96.44 nm, with a median particle size (D50) of 75.8 nm and a particle size (D90) of 81.44 nm, and the overall size of poly(VS-St-BMA-BA) is in the nanometer range and can be used as a nano-plugging agent.

### 2.3 Thermogravimetric Analysis

**Figure 3:**
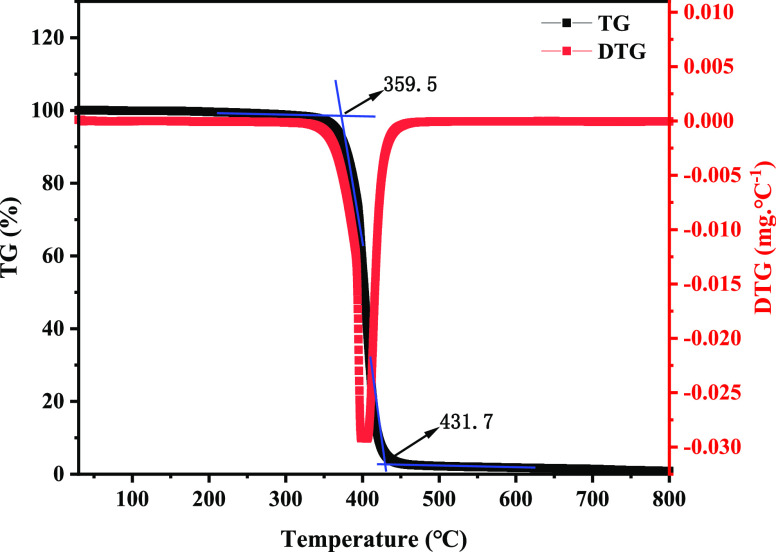
Poly(VS-St-BMA-BA) thermogravimetric analysis chart.

Actual shale reservoirs usually have high temperatures, and temperature resistance is an extremely important influence on the sealing performance of nanomaterials. Figure 3 shows the thermal weight loss analysis curves of the nano-plugging agent poly(VS-St-BMA-BA). From the TG-DTG curve in Figure 3, it can be seen that there is a small decrease (3.92%) in the thermal weight loss curve in the range of 102.0 to 359.5 °C; this part of the lost mass is caused by the evaporation of the bound water in the plugging agent. The initial decomposition temperature of the nano-plugging agent poly(VS-St-BMA-BA) was 359.5 °C, and when the temperature reached 431.7 °C, the mass loss from 359.5 to 431.7 °C was 91.03%, and the basic thermal decomposition of poly(VS-St-BMA-BA) was completed. It indicates that the synthesized nano-plugging agent poly(VS-St-BMA-BA) has good temperature resistance. The structure of poly(VS-St-BMA-BA) contains several benzene rings with strong temperature resistance, and the two S–O (π-bonds) bonding liquids in the sulfonic acid group also have good temperature resistance, so poly(VS-St-BMA-BA) can resist high temperatures above 300 °C and has excellent high-temperature stability.

### 2.4 High-Temperature Stability

**Figure 4:**
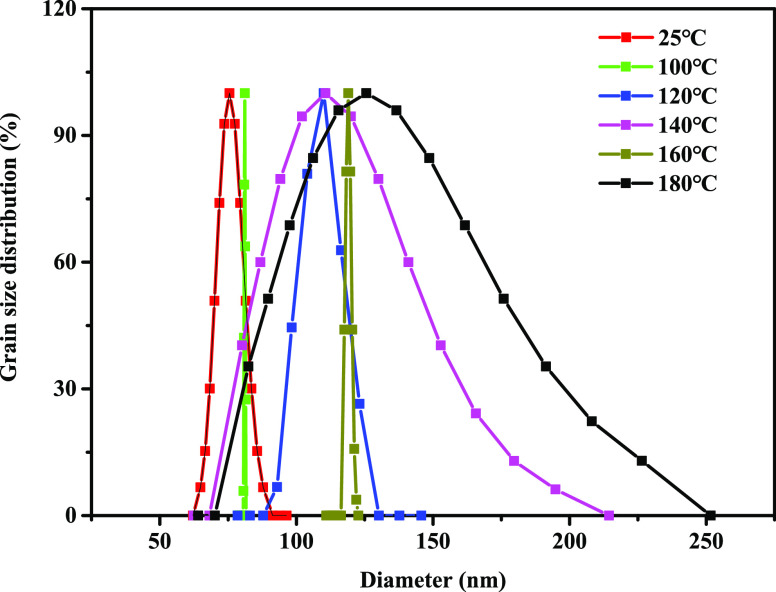
Particle size distribution of poly(VS-St-BMA-BA) after different temperature treatment.

The excellent dispersion stability of poly(VS-St-BMA-BA) at high temperature is the key to achieve effective sealing of shale nanopores. In order to investigate the dispersion stability of poly(VS-St-BMA-BA) at high temperature, the change in the particle size of the plugging agent after high-temperature treatment can effectively derive the temperature resistance of the plugging agent and its dispersion stability performance at high temperature. The particle size distribution of poly(VS-St-BMA-BA) before and after aging at different temperatures is shown in Figure 4.

From Figure 4, the median particle size D50 of poly(VS-St-BMA-BA) was 75.8 nm at room temperature, and the particle size distribution ranged from 62.17 to 96.44 nm. After aging at 100 °C, the median particle size D50 of the plugging agent (81.1 nm) increased a little, and the particle size distribution ranged from (117.55 and 121.94 nm). After aging at 120 °C, the median particle size D50 was 100 nm, and the particle size distribution was 87.8–130.2 nm. After aging at 140 °C, the median particle size D50 was 85 nm, the particle size distribution was 68.2–214.4 nm, and the distribution range became wider. After aging at 160 °C, the median particle size was 117 nm, and the particle size distribution was between 116.4 and 122.7 nm. After aging at 180 °C, the particle size range was between 70.1 and 251.6 nm, and the distribution range changed a lot, but the median particle size D50 was 89.7 nm, which was not much changed compared with the median particle size under normal temperature. Under the high-temperature conditions of 100, 120, 140, 160, and 180 °C, the plugging agent had good anti-high-temperature stability, and the median particle size did not change significantly, which confirmed that poly(VS-St-BMA-BA) has good high-temperature resistance and excellent dispersion stability under extreme high-temperature environment.

### 2.5 Drilling Fluid Performance Evaluation

**Figure 5:**
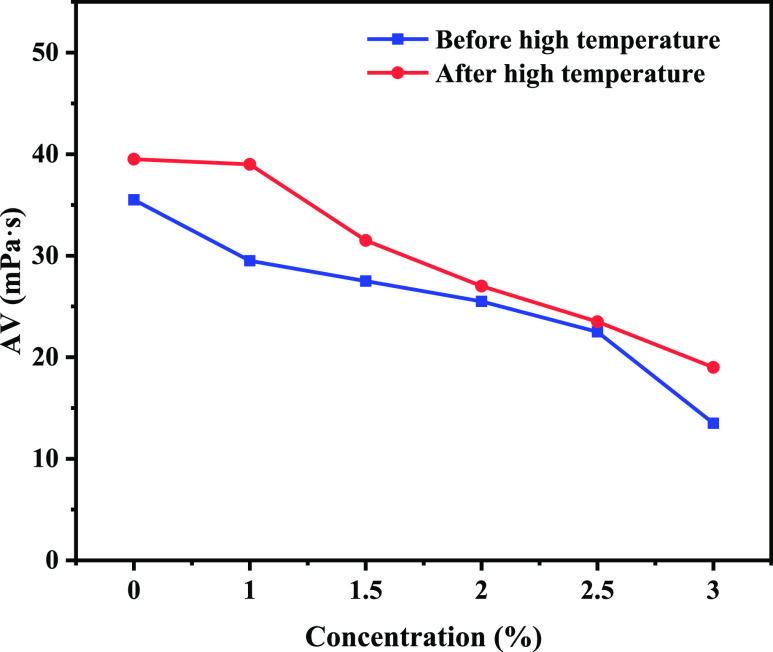
AV change curve of water-based drilling fluid with the addition of poly(VS-St-BMA-BA) before and after aging.

**Figure 6:**
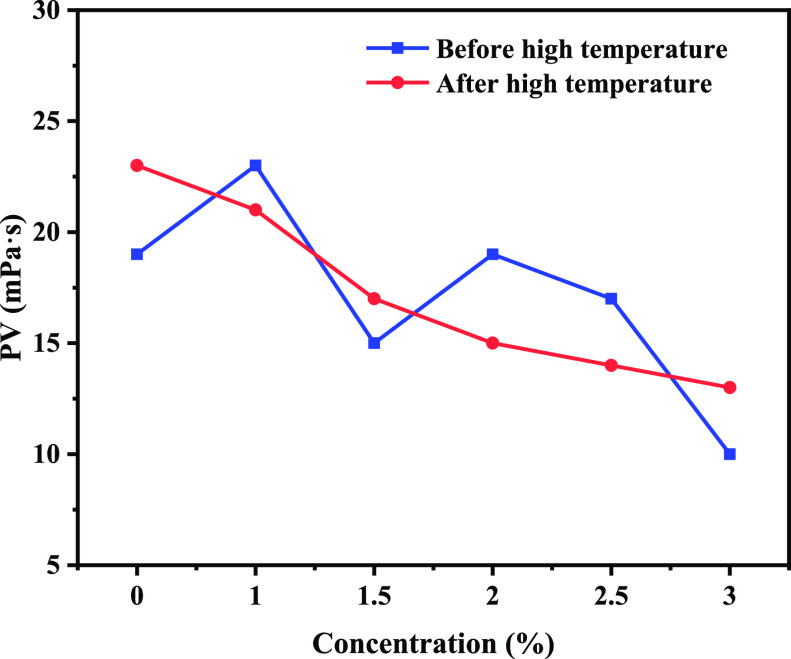
PV change curve of water-based drilling fluid with the addition of poly(VS-St-BMA-BA) before and after aging.

**Figure 7:**
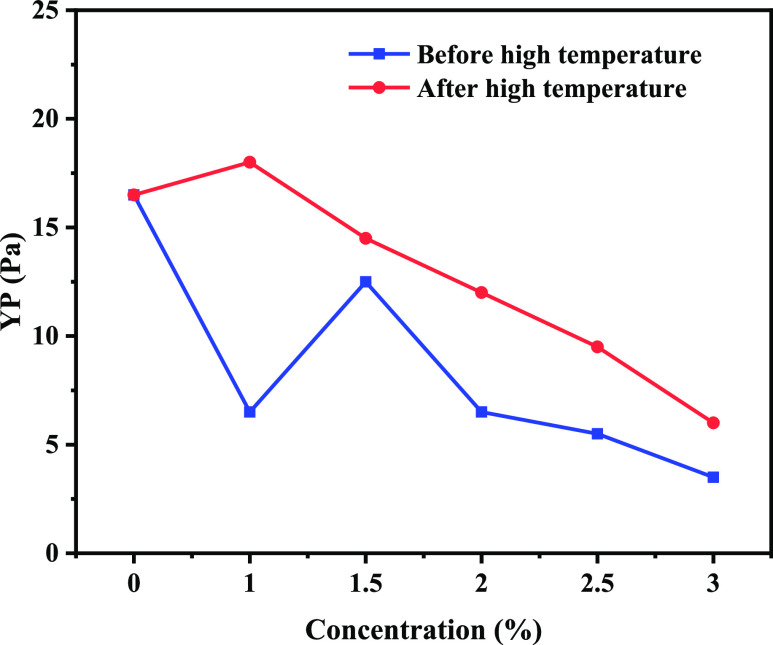
Change curve of YP of water-based drilling fluid with the addition of poly(VS-St-BMA-BA) before and after aging.

**Figure 8:**
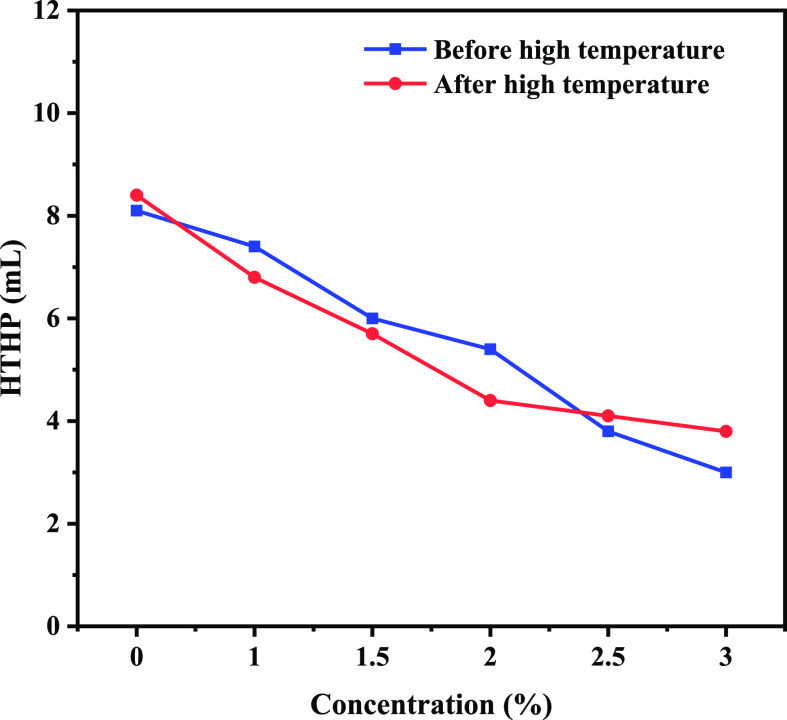
HTHP curve of water-based drilling fluid before and after aging after adding poly(VS-St-BMA-BA).

The drilling fluid base slurry was added with 1.0, 1.5, 2.0, 2.5, and 3.0% concentration of poly(VS-St-BMA-BA) and aged at 150 °C for 16 h. The drilling fluid properties before and after aging are shown in Figures 5, 6, 7, and 8.

#### 2.5.1 Apparent Viscosity

Figure 5 shows the apparent viscosity (AV) change curve of drilling fluid. From Figure 5, it can be seen that the AV of drilling fluid decreases with the increase in poly(VS-St-BMA-BA) addition before aging. The AV of the drilling fluid base slurry was 35.5 mPa·s. The corresponding AVs of the drilling fluid were 29.5, 27.5, 25.5, 19.0, 13.5, and 13.5 mPa·s for the addition of poly(VS-St-BMA-BA) at 1.0–3.0% (1.0, 1.5, 2.0, 2.5, and 3.0%). The AV of drilling fluid decreased by 61.97% compared with that of drilling fluid base slurry when poly(VS-St-BMA-BA) was added at 3.0%, and a significant decrease occurred. Poly(VS-St-BMA-BA) addition had a large impact on the AV of drilling fluid. The AV of the drilling fluid base slurry was 39.5 mPa·s after high-temperature aging at 150 °C for 16 h. The corresponding AVs of drilling fluid were 39.0, 31.0, 27.0, 23.5, and 19.0 mPa·s when the addition amount of poly(VS-St-BMA-BA) was 1.0–3.0%. The AV of drilling fluid increased compared with that before aging, and the change trend was similar to that before aging. Poly(VS-St-BMA-BA) showed a decreasing trend in AV before and after aging to different degrees, which is due to the fact that the poly(VS-St-BMA-BA) nano-plugging agent can increase the electric potential of clay particles and the thickness of hydration films, increase the inter-particle repulsion, and dismantle the drilling fluid in the inter-particle mesh structure of clay, which has a certain effect of viscosity reduction.

#### 2.5.2 Plastic Viscosity

Figure 6 shows the plastic viscosity (PV) change curve of drilling fluid. As can be seen from Figure 6, the PV of the drilling fluid before aging showed a decreasing trend with the increase in the addition of poly(VS-St-BMA-BA). The PV of the drilling fluid base slurry was 19.0 mPa·s, and the corresponding PVs of the drilling fluid were 23.0, 15.0, 19.0, 17.0, and 10.0 mPa·s when the addition amount of poly(VS-St-BMA-BA) was 1.0–3.0%. The PV of the drilling fluid base slurry was 23.0 mPa·s after aging at 150 °C for 16 h. The PVs of the corresponding drilling fluids were 21.0, 17.0, 15.0, 14.0, and 13.0 mPa·s when the dosage of poly(VS-St-BMA-BA) was 1.0–3.0%. The SMC (sulfonated lignite) and SMP-1 (sulfonated phenol formaldehyde resin) treatment agents in drilling fluids will give full play to their effects only after high-temperature aging, so the change pattern of PV of drilling fluids before aging is not obvious. The PV of the drilling fluid did not change much compared to that before aging, and the changes were more regular. The PV of drilling fluids with the addition of poly(VS-St-BMA-BA) also showed a decreasing trend before and after aging, which was due to the strong adsorption ability of poly(VS-St-BMA-BA) nano-sealers, which competed for participation in clay-polymer adsorption sites in water-based drilling fluids, dismantled the clay-polymer mesh structure in drilling fluids, and reduced the PV of drilling fluids.

#### 2.5.3 Yield Point

Figure 7 shows the yield point (YP) change curve of drilling fluid. The YP of drilling fluid base slurry before aging is 16.5 Pa. When the amount of poly(VS-St-BMA-BA) is 1.0–3.0%, the corresponding YPs are 6.5, 12.5, 6.5, 5.5, and 3.5 Pa. The YP decreases significantly with the increase in poly(VS-St-BMA-BA) addition, which has a greater impact on the rock-carrying performance of drilling fluid. The YP of the drilling fluid base slurry was 16.5 Pa after high-temperature aging at 150 °C for 16 h. The corresponding YPs for drilling fluids with 1.0 to 3.0% addition were 18.0, 14.5, 12.0, 9.5, and 6.0 Pa. Compared with before aging, the YP of drilling fluid increased after aging due to the effect of drilling fluid treatment agents after high-temperature aging and decreased with poly(VS-St-BMA-BA) addition.

#### 2.5.4 High Temperature and High Pressure Water Loss

Figure 8 shows the high temperature and high pressure (HTHP) variation curve of drilling fluid. From Figure 8, it can be seen that the water loss of drilling fluid decreases with the increase in poly(VS-St-BMA-BA) addition. Before aging, the HTHP of drilling fluid base slurry was 8.1 mL. the corresponding HTHP was 7.4, 6.0, 5.4, 3.8, and 3.0 mL for the addition of poly(VS-St-BMA-BA) from 1.0 to 3.0%. After aging at 150 °C, the HTHP of drilling fluid base slurry was 8.4 mL. poly(VS St-BMA-BA) was added at 1.0 to 3.0%, corresponding to HTHP of 6.8, 5.7, 4.4, 4.1, and 3.8 mL, similar to the trend before aging.

#### 2.5.5 Summary

In general, HTHP gradually decreases with the increase in poly(VS-St-BMA-BA) addition, and the addition of poly(VS-St-BMA-BA) has a certain influence on the rheological parameters of drilling fluid. When the addition of poly(VS-St-BMA-BA) exceeded 2.0%, the decreasing trend of HTHP of the aging drilling fluid became slower, therefore combining the AV and PV yield. Therefore, the overall performance of the drilling fluid after aging was better when the amount of poly(VS-St-BMA-BA) was added at 2%, which combined the AV, PV, YP, and HTHP of the drilling fluid.

### 2.6 Artificial Mud Cake Sealing Performance Evaluation

**Figure 9:**
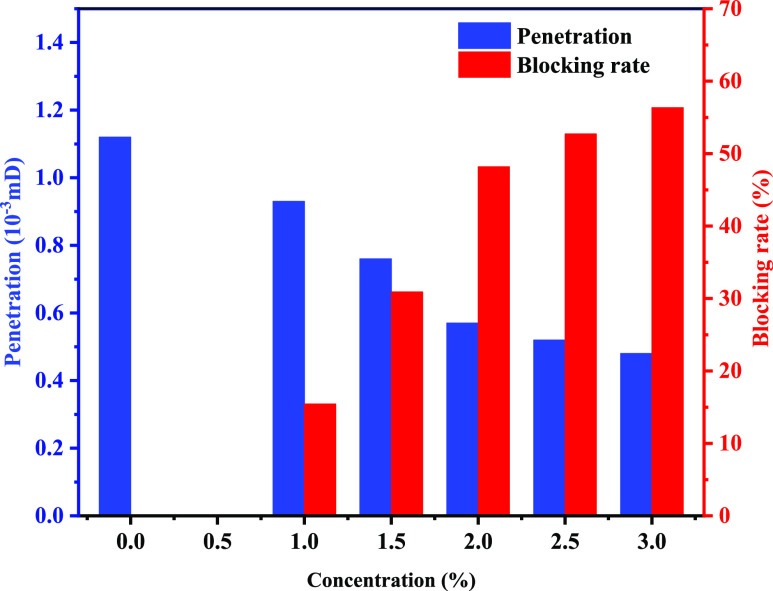
Variation of permeability and the plugging rate of poly(VS-St-BMA-BA) with different dosages.

The evaluation of the sealing effect of poly(VS-St-BMA-BA) on artificial mud cake at 150 °C and a differential pressure of 3.5 MPa is shown in Figure 9. As can be seen from Figure 9, the permeability of the artificial mud cake without plugging agent is 1.12 × 10^–3^ mD at 150 °C and 3.5 MPa pressure difference, which is a permeability class of 10^–3^ mD, close to the shale permeability, and can be used to simulate the evaluation of the plugging performance of shale. After the addition of poly(VS-St-BMA-BA), the permeability of the artificial mud cake decreased with the increase in poly(VS-St-BMA-BA), and the sealing rate increased gradually with the increase in the addition, When the addition of poly(VS-St-BMA-BA) reached 2%, the permeability was reduced to 0.57 × 10^–3^ mD, and the plugging rate was 48.18% at this time. As the addition continues to increase to 2.5 and 3%, the plugging rate rises to 52.73 and 56.36%, and although there is some increase, the change is not significant. Therefore, considering the combination of the sealing effect and economic benefits, the best dosage of poly(VS-St-BMA-BA) nano-plugging agent is 2%, and the drilling fluid has excellent nano-plugging ability when the dosage of poly(VS-St-BMA-BA) is 2%.

### 2.7 Artificial Core Sealing Performance Evaluation

**Figure 10:**
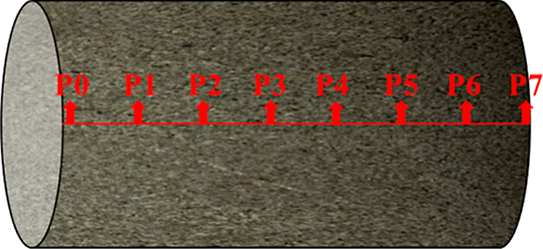
Schematic diagram of pressure sensor location.

From the inlet to the outlet, P0 is the sensor at 2 mm of the artificial core inlet, and the remaining consecutive pressure sensors P1, P2, P3, P4, P5, P6, and P7 are used to record the pressure data of each part of the fluid as it passes through the core (Figure 10). Due to the abnormal data from the P2 pressure sensor, the experiments were analyzed using the data recorded by P0, P1, P3, P4, P5, P6, and P7.

**Figure 11:**
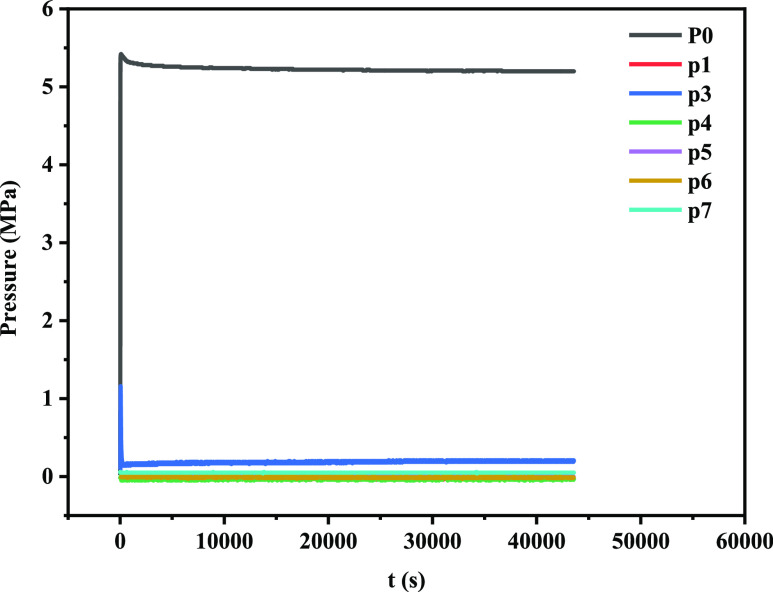
Poly(**VS**-St-BMA-BA) plugging artificial core pressure transfer diagram.

In order to verify the sealing performance and sealing position of poly(VS-St-BMA-BA) in artificial rock cores under high temperature and pressure conditions, pressure-transfer experiments were conducted at a temperature of 105 °C and an inlet pressure of 5.5 MPa. As can be seen from Figure 11, after adding 2% poly (VS-St-BMA-BA), the pressure at P0 increased rapidly to about 5.4 MPa, and there was almost no pressure at P1, P4, P5, P6, and P7, but the pressure value at P3 increased suddenly at the beginning and then decreased gradually until it decreased to 0.2 MPa. At P0, the pressure becomes larger; thus, it is clear that the nano-sealer poly (VS-St-BMA-BA) has sealed to the core surface fracture of about 2 mm at P0. However, a very small amount of the filtrate still enters the interior of the core and then fails rapidly in a high temperature and pressure environment, with the result that the pressure at P3 suddenly increases and then stabilizes at a smaller pressure value. The above experiment shows that the plugging agent forms an effective plugging at P0.

The sealing evaluation of the artificial cores at a temperature of 105 °C and a pressure difference of 3.5 MPa is shown in Table 1. From Table 1, it can be seen that when the optimum addition amount of poly(VS-St-BMA-BA) solution of 2% was added to the artificial core apparatus, the sealing rate was as high as 88.75%, indicating that poly(VS-St-BMA-BA) has good sealing performance. Combined with pressure-transfer experiments, it is known that poly(VS-St-BMA-BA) can form a very effective seal at a certain distance of nanopore joints on the surface of artificial cores.

**Table 1:** Evaluation of the Sealing Effect of Poly(VS-St-BMA-BA) on Artificial Cores at 105 °C

name	permeability after plugging/10^–3^ mD	plugging rate/%
clearwater	5.78	
2% poly(VS-St-BMA-BA) + clearwater	0.65	88.75

### 2.8 Micromorphological Studies

**Figure 12:**
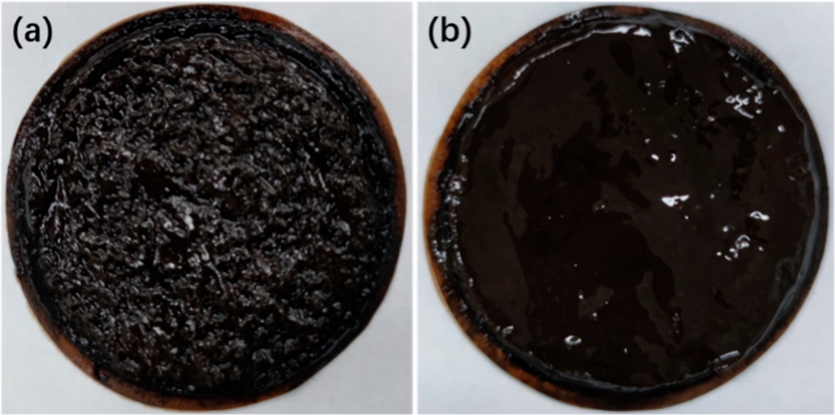
Macroscopic morphology of unsealed artificial mud cake and poly(VS-St-BMA-BA)-sealed artificial mud cake; (a) unsealed artificial mud cake and (b) poly(VS-St-BMA-BA)-sealed artificial mud cake.

**Figure 13:**
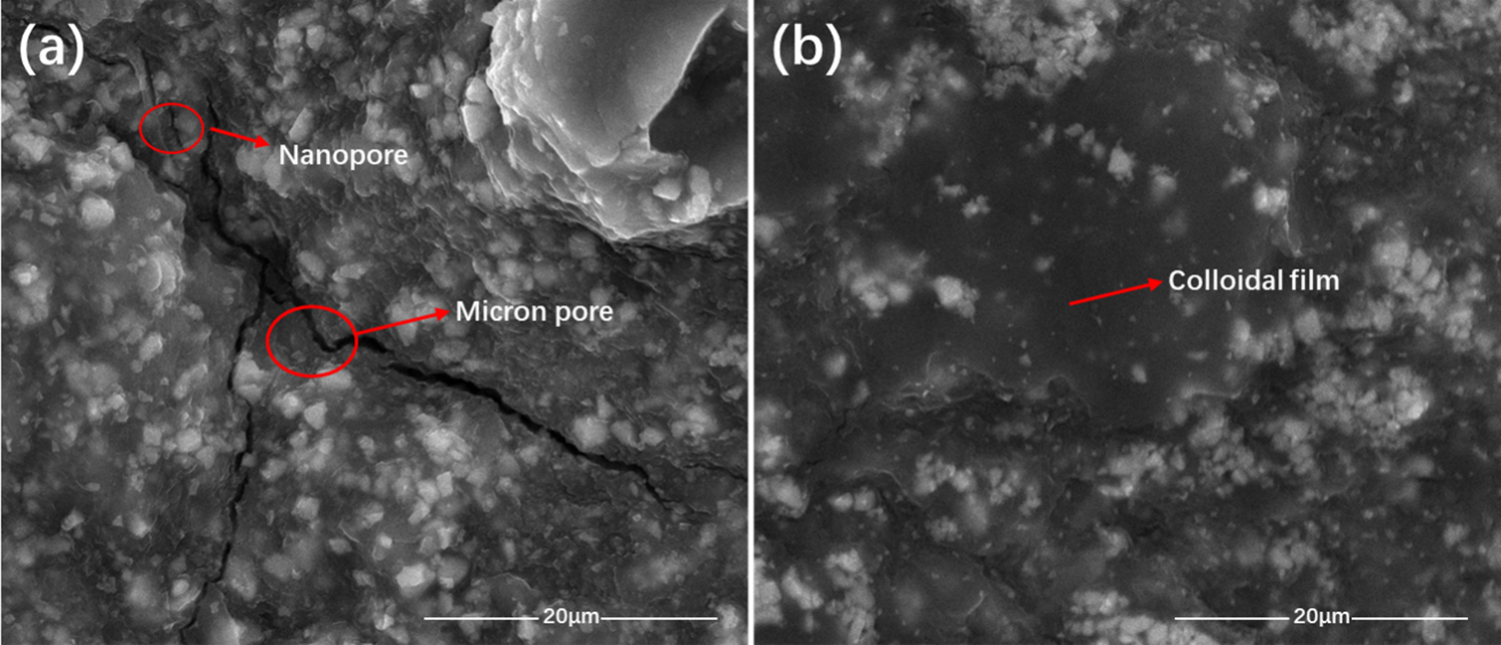
Scanning electron micrographs of artificial mud cake before plugging and poly(VS-St-BMA-BA) after plugging; (a) artificial mud cake before plugging and (b) poly(VS-St-BMA-BA) after plugging.

The surface macroscopic morphology and electron microscopic scanning microstructure of the unblocked artificial mud cake and poly(VS-St-BMA-BA) blocked artificial mud cake are shown in Figures 12 and 13.

The macroscopic morphology of the unblocked artificial mud cake is shown in Figure 12a. From Figure 12a, it can be seen that the surface of the artificial mud cake with the plugging agent is uneven, with a large number of pores and gullies. The macroscopic morphology of the artificial mud cake blocked by poly(VS-St-BMA-BA) is shown in Figure 12b, and it can be seen from Figure 12b that the surface of the artificial mud cake blocked by poly(VS-St-BMA-BA) is flat and smooth, and the surface density is significantly better than that of the unblocked artificial mud cake, as shown in Figure 12a.

The microscopic morphology of the low-permeability artificial mud cake without and with 2% poly (VS-St-BMA-BA) plugging is shown in Figure 13. The unblocked artificial mud cake is shown in Figure 13a. From Figure 13a, it can be seen that the surface electron micrograph of the unblocked mud cake shows a large number of micro- and nanopore slits, whose pore slit widths ranging from tens of nanometers to several microns, and these micro- and nanopore slits can be used as seepage channels for the drilling fluid filtrate to invade the well wall, which in turn leads to well wall destabilization. The microscopic morphology of the artificial mud cake after plugging by poly(VS-St-BMA-BA) is shown in Figure 13b, which shows that the surface microscopic morphology of the mud cake after plugging is flat and dense without the appearance of pores and seams, and the surface is covered with a transparent film, forming a dense nano-plugging layer on the surface of the artificial mud cake with good plugging effect.

Based on the macroscopic morphology and microstructure analysis, the excellent sealing performance of poly(VS-St-BMA-BA) on micro- and nanopores under extreme conditions of high temperature and pressure is demonstrated.

### 2.9 Nano Plugging Mechanism Research

**Figure 14:**
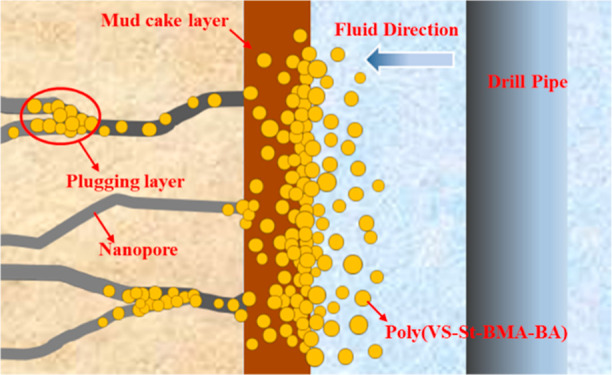
Diagram of the poly(VS-St-BMA-BA) plugging mechanism.

Figure 14 shows the sealing mechanism of poly(VS-St-BMA-BA) in microfracture developed formations. Poly(VS-St-BMA-BA), as a nano-plugging agent, has hydrophilic sulfonic acid groups and hydrophobic ester groups in its structure, where the hydrophilic groups are attached to the hydrophilic component of the clay and the hydrophobic part (containing ester groups) forms a colloidal film on the wellbore wall, thus reducing the filtrate intrusion.^26^ During the process of filter cake formation, a portion of poly(VS-St-BMA-BA) passes through the filter cake with the filtrate. When the size of poly(VS-St-BMA-BA) is larger than the size of the nanopore, poly(VS-St-BMA-BA) may bridge and stack at the entrance of nanopore. When the size is close to or smaller than that, poly(VS-St-BMA-BA) will enter the nanopore in the formation along the fluid direction and accumulate into a plugging layer at the depth to further reduce the filtrate intrusion.^16^ As the amount of poly(VS-St-BMA-BA) increases, the faster the colloidal film formed and the tighter the buildup, the less the amount of filtrate will be.

This study shows that the prepared modified poly(VS-St-BMA-BA) can be used as a high-performance nano-plugging agent in water-based drilling fluids to effectively reduce the intrusion of drilling fluids into the microporous joints of the formation and prevent the weakening of rock strength, thus playing a role in protecting the reservoir.

## 3 Conclusions

(1) Polymer nanoparticles poly(VS-St-BMA-BA) with a median particle size D50 of 75.8 nm were synthesized from sodium vinyl sulfonate, styrene, BMA, and BA. Poly(VS-St-BMA-BA) nanoparticles can resist 359.5 °C high temperature and can be well dispersed in water-based drilling fluid, which can effectively improve the sealing ability of drilling fluid.
(2) Poly(VS-St-BMA-BA) has less effect on the performance of water-based drilling fluids, and the optimal addition amount is 2%. After aging at 150 °C, the drilling fluid with 2% poly(VS-St-BMA-BA) nano-plugging agent added had a YP of 12 Pa, a HTHP of 4.4 mL, a good rock carrying capacity and plugging capacity, and excellent drilling fluid performance. Poly(VS-St-BMA-BA) can resist 359.5 °C high temperature, and the sealing rate of artificial mud cake increases gradually with the increase in poly(VS-St-BMA-BA) addition. When the addition amount is 2%, the sealing rate is 48.18%, and the sealing rate in the artificial core experiment, when the addition amount of poly(VS-St-BMA-BA) is 2%, the sealing rate reaches 88.75%. From the pressure-transfer experiment, it can be seen that the main plugging location is 2 mm from the pore slit on the surface of the artificial core. The performance of drilling fluid with poly(VS-St-BMA-BA) is more stable, and the artificial mud cake filtration loss test and artificial core penetration test both show that poly(VS-St-BMA-BA) has excellent plugging effects.
(3) Poly (VS-St-BMA-BA), as a nanomaterial, can enter the nanopores under formation pressure and continuously accumulate to form a bridge, forming an effective sealing layer. Poly(VS-St-BMA-BA) has strong adsorption property and can be adsorbed on the surface of filter cake to form a colloidal film to prevent drilling fluid filtrate from penetrating into the shale formation. Poly(VS-St-BMA-BA) is expected to be a potential nano-plugging agent for water-based drilling fluids.

## 4 Materials and Methods

### 4.1 Materials

Sodium dodecyl sulfate (SDS), sodium VS, styrene (St), BMA, BA, DVB, ammonium persulfate (APS), and anhydrous Na_2_CO_3_ were obtained from Chengdu Kelong Chemical Reagent Factory; anti-temperature and anti-salt filter loss reducing agent (HF-1), filter loss reducing agent (SMC), filter loss reducing agent (SMP-1), and millimicron barite powder were obtained from Sichuan Southwest Shida Jinniu Technology Co; bentonite was obtained from Xinjiang China Central Africa Xiazi Street Bentonite Co, Ltd; and an artificial core was obtained from Chengdu Keping Technology Co.

### 4.2 Instruments

Laser Scattering System (BI-200SM) was obtained from Brookhaven Instruments, USA; FTIR spectrometer (Nicolet 6700) was obtained from American Thermoelectric Corporation; synchronous thermal analyzer (TGA/DCS1) was obtained from MATTLER, Switzerland; HTHP filter loss instrument (GGS42-2A) was obtained from Shandong Qingdao Haitongda Special Instrument Co; six-speed rotational viscometer (1103) was obtained from Shandong Meike Instruments Co; environmental scanning electron microscope (Quanta450) was obtained from FEI, USA; and SCMS-C4 HTHP dense core permeability testing device (20172034-1) was obtained from Chengdu Haohan Completion Rock & Electric Technology Co.

### 4.3 Preparation of Nanomaterials

A small amount of SDS and VS is weighed into a three-necked flask, ultrapure water was added to dissolve them, appropriate amounts of St, BMA, BA, and DVB were added, the three-necked flask was placed in a water bath, stirred at 300 rpm, heated to the reaction temperature, nitrogen was passed, and ultrapure water containing APS was added after 20 min and reacted for 8 h to obtain poly(VS-St-BMA-BA).

### 4.4 High-Temperature Stability

The temperature stability of the nanoparticles was determined by examining the size of the nanoparticle particle size of the poly(VS-St-BMA-BA) nano-plugging agent after treatment at different temperatures. Equal amounts of poly(VS-St-BMA-BA) with 1% mass concentration were aged at 100, 120, 140, 160, and 180 °C for 16 h, respectively. The particle size of poly(VS-St-BMA-BA) dispersions at different aging temperatures was measured using a laser scattering system (BI-200SM) after 24 h resting, the dispersion stability of poly(VS-St-BMA-BA) at different temperatures was investigated based on the measured particle size data.

### 4.5 Artificial Mud Cake High Temperature and High Pressure Water Loss

The drilling fluid base slurry made in Section 2.4 was poured into the high-temperature and high-pressure filter loss apparatus, and the mud cake was pressed for 30 min at 150 °C and a differential pressure of 3.5 MPa to obtain an artificial mud cake. Different mass concentrations (1, 1.5, 2, 2.5, and 3%) of poly(VS-St-BMA-BA) solution were prepared separately. The artificial mud cakes were loaded into the HTHP water loss meter, respectively; first, the water is flowed through the HTHP water loss meter at 150 °C, and the pressure difference is 3.5 MPa to test the artificial mud cake permeability, then different amounts of poly(VS-St-BMA-BA) were added into the HTHP water loss meter with artificial mud cake, and the sealing performance was tested at 150 °C and 3.5 MPa differential pressure. During the test, the filtrate was collected at 5 min intervals, and the volume of the filtrate was recorded and maintained for 30 min. When the experiment was completed, the thickness of the filter cake was measured, and the permeability of the filter cake after the addition of poly(VS-St-BMA-BA) solution was calculated using eq 1 based on Darcy’s law
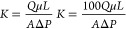
1

In the formula: K—permeability of the “artificial mud cake”, mD; *Q*—average volume of water loss per second, cm^3^/s; μ—viscosity of the filtrate, mPa·s; *L*—thickness (or length) of the mud cake, cm; *A*—area of the filter cake, and cm^2^; Δ*P*—filter loss differential pressure, MPa.

### 4.6 Artificial Rock Core Permeation Experiment

The poly(VS-St-BMA-BA) was prepared as a 300 mL solution at 2.5% mass concentration of the optimal plugging effect and then ultrasonically dispersed at a temperature of 70 °C for 30 min. Water and poly(VS-St-BMA-BA) solution were added to the SCMS-C4 high-temperature and high-pressure dense core permeability testing device, and the artificial core permeability test experiment was conducted at 105 °C with a pressure difference of 3.5 MPa. Calculation of artificial core permeability, that is, the formula for calculating the permeability is shown in eq 1 and described in Section 4.5.
